# An Unusual Presentation of Frontal Pneumosinus Dilatans as Flight-Related Headache With Favorable Response to Carbamazepine

**DOI:** 10.7759/cureus.113052

**Published:** 2026-07-20

**Authors:** Sumeet Angral, Divyanshi Singh, Ritu Raj, Sandeep Sehrawat

**Affiliations:** 1 Otolaryngology, All India Institute of Medical Sciences, Bilaspur, Bilaspur, IND; 2 Otolaryngology - Head and Neck Surgery, All India Institute of Medical Sciences, Bilaspur, Bilaspur, IND; 3 Internal Medicine, All India Institute of Medical Sciences, Jammu, Jammu, IND; 4 Orthopaedics, All India Institute of Medical Sciences, New Delhi, New Delhi, IND

**Keywords:** barosinusitis, frontal sinus, neuralgia, paranasal sinus disease, pneumosinus dilatans frontalis

## Abstract

Pneumosinus dilatans (PSD) is an uncommon disorder marked by an abnormal hyperpneumatization of a paranasal sinus with intact bony walls. Although it is usually asymptomatic, it can cause headaches or other pressure-related symptoms, especially during changes in barometric pressure. We report a case of isolated frontal PSD in a 28-year-old man who complained of recurring, pressure-like frontal headaches that only occurred during air travel. The clinical examination, diagnostic nasal endoscopy, and neurological assessment were unremarkable. High-resolution computed tomography indicated hyperaeration of the frontal sinus with intact bony walls, which is consistent with PSD frontalis. Conventional treatments for barosinusitis, such as topical and systemic decongestants, were ineffective. Given a probable neuropathic pain mechanism caused by altitude-related pressure changes, carbamazepine medication was started, resulting in considerable symptomatic improvement and long-term pain relief at six months. The present case demonstrates PSD as an unusual but relevant differential diagnosis in flight-induced headaches, and it suggests that neuropathic pain modulators may play a role in certain patients.

## Introduction

Pneumosinus dilatans (PSD) is a rare pathological condition characterized by hyperpneumatization of one or more paranasal sinuses, resulting in deformation and displacement of the surrounding bony and soft tissues, leading to varying signs and symptoms. The frontal sinus is the most frequently involved sinus, followed by the sphenoid, maxillary, and ethmoid sinuses [1].

Though often asymptomatic, PSD can present with facial asymmetry, visual disturbances, or headache, symptoms that may be influenced by changes in barometric pressure, such as during flights [2].

Barometric headaches are commonly attributed to barosinusitis or migraine; however, when standard treatments such as decongestants fail, rare anatomical abnormalities like PSD should be considered. We present a case of isolated frontal PSD in a young man with flight-induced headaches, highlighting the diagnostic challenges and management approach.

## Case presentation

A 28-year-old male patient presented to our outpatient department with complaints of recurrent bilateral frontal headaches that occurred exclusively during air travel, with no prior history of nasal obstruction, nasal discharge, excessive sneezing, nasal bleed, allergic rhinitis, nasal surgery, facial trauma, sinusitis, phonophobia, giddiness, epilepsy, migraine, chronic analgesic use, or neurological disorder. The patient was a frequent flyer as he worked overseas and had to travel frequently for his professional engagements. Symptoms began shortly after takeoff and worsened with longer flights (>4 hours), resolving spontaneously a few hours post-landing. The headaches were described as pressure-like, non-pulsatile, localized to the forehead, and were not accompanied by nasal discharge, congestion, fever, photophobia, or aura.

On diagnostic nasal endoscopy, there were no abnormalities. A trial of topical and systemic nasal decongestants (oxymetazoline and pseudoephedrine) prior to flights provided no relief. Both ears were normal on otoscopic examination. Neurological examination was unremarkable.

**Figure 1:**
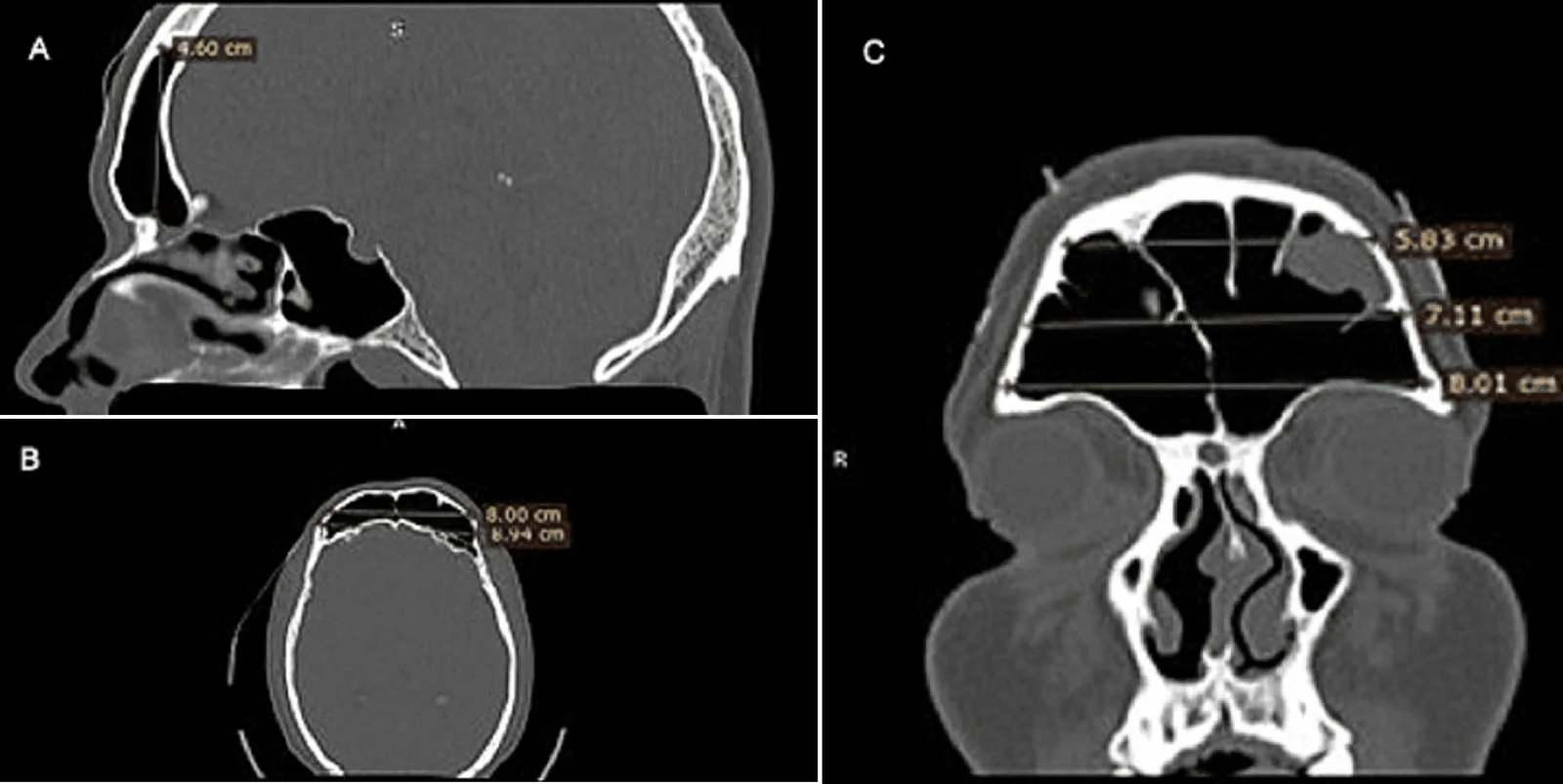
Non-contrast CT scan of nose and paranasal sinuses depicting pneumosinus dilatans (A) Sagittal view showing craniocaudal dimension of 4.60 cm; (B) Axial view showing average transverse dimension of 8.47 cm, and (C) Coronal view depicting average transverse dimension of 6.98 cm of the frontal sinus.

**Figure 2:**
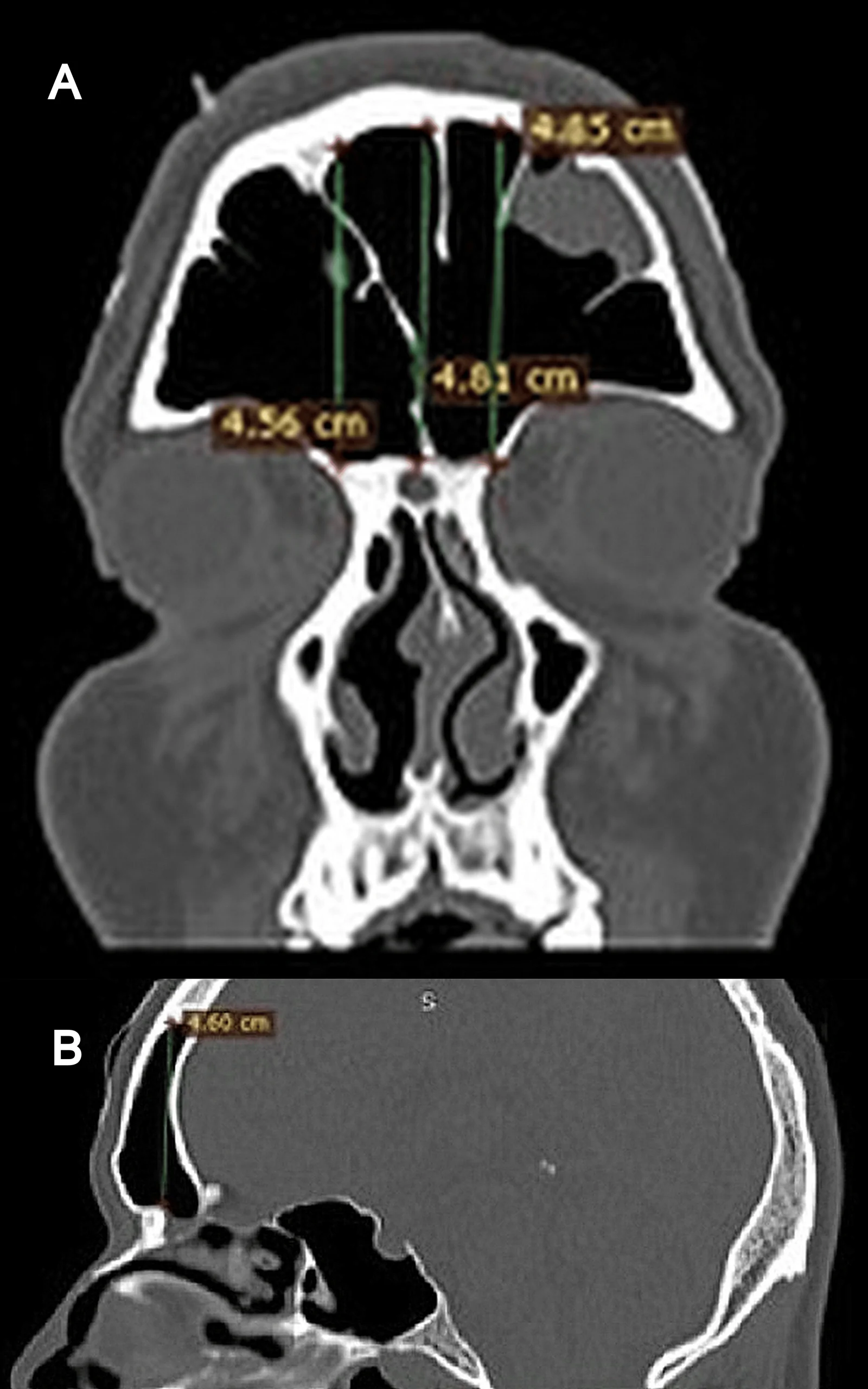
Non-contrast CT scan of nose and paranasal sinuses depicting neumosinus dilatans (A) Coronal view showing average craniocaudal dimension of 4.74 cm and (B) Sagittal view showing craniocaudal dimension of  4.60 cm of the frontal sinus

**Figure 3:**
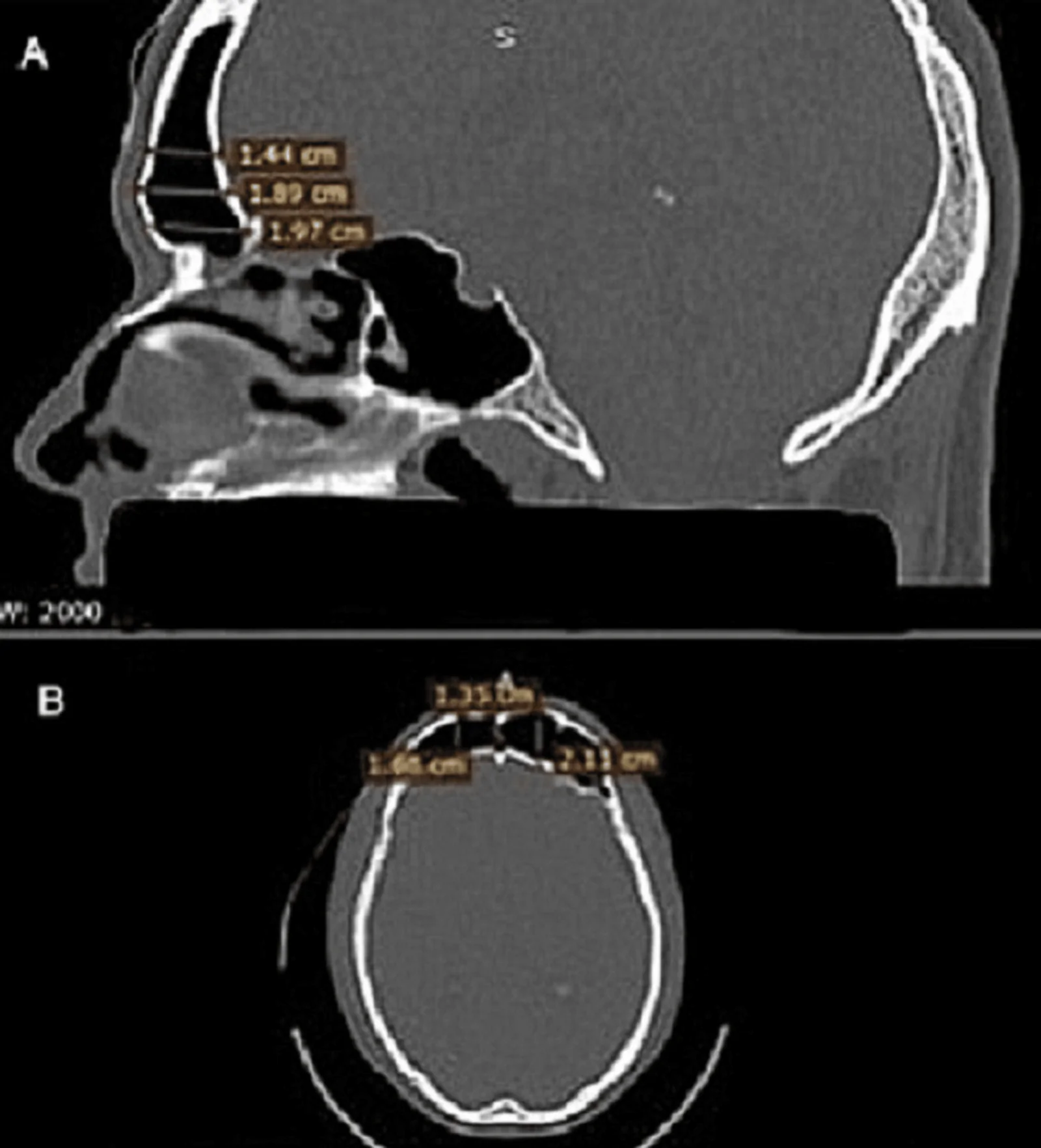
Non-contrast CT scan of nose and paranasal sinuses depicting pneumosinus dilatans (A) Sagittal view showing average antero-posterior dimension of 1.76 cm and (B) Axial view depicting average anteroposterior dimension of  1.71 cm of frontal sinus

High-resolution computed tomography (CT) of the paranasal sinuses revealed marked expansion of the frontal sinus beyond the normal anatomical boundaries with intact bony margins and minimal mucosal thickening (mainly in the superior part of the left frontal sinus region), findings consistent with PSD frontalis. The dimensions were measured at the widest part of the sinus, and the average was taken to measure the dimensions in sagittal, axial, and coronal planes. Figure 1A shows a sagittal view depicting a craniocaudal dimension of 4.60 cm; Figure 1B shows an axial view depicting an average transverse dimension of 8.47 cm, and Figure 1C shows a coronal view depicting an average transverse dimension of 6.98 cm of the frontal sinus. Figure 2A shows a coronal view depicting an average craniocaudal dimension of 4.74 cm, and Figure 2B shows a sagittal view depicting a craniocaudal dimension of 4.60 cm of the frontal sinus. Figure 3A shows a sagittal view depicting an average anteroposterior dimension of 1.76 cm, and Figure 3B shows an axial view depicting an average anteroposterior dimension of 1.71 cm of the frontal sinus.

The patient was not subjected to magnetic resonance imaging (MRI) because CT demonstrated isolated disease. Given the failure of decongestants and suspicion of a neuropathic pain mechanism triggered by barometric pressure change, a trial of carbamazepine 100 mg twice daily was initiated after baseline complete blood count, liver function tests, and serum sodium were normal and continued for a period of four weeks. The patient was counseled regarding the side effects.

The patient reported significant improvement in symptom frequency and intensity over the next two flights and continued the medication with good tolerance. At the six-month follow-up, he remained stable and reported that there was symptomatic relief from symptoms on taking carbamazepine before boarding the flight and there was a reduction in flight-related headache episodes.

## Discussion

PSD is characterized by the enlargement of an aerated sinus beyond normal wall borders in the absence of regional bone damage, hyperostosis, or mucous membrane thickening [2]. A significant proportion of patients only present with cosmetic concerns or are diagnosed on radiology alone as they are asymptomatic; thus, it is safe to assume that the true incidence may be much higher than anticipated [3]. There is a difference in PSD prevalence related to gender specificity, and it is considered that the occurrence in men is almost twice as common as in women, with a peak incidence between 20 and 40 years of age [2]. The etiology of PSD remains unclear but has been associated with various conditions like anterior skull base or optic nerve sheath meningiomas, arachnoid cysts, or fibro-osseous disorders [4-6].

The pathogenesis of PSD remains poorly understood, although several hypotheses have been proposed. These include the "ball-valve" mechanism, in which a one-way air valve effect leads to progressive air trapping and sinus expansion, increased local gas production by bacteria, and congenital or developmental anomalies leading to impaired ventilation [7]. Radiologically, PSD is characterized by a hyperaerated sinus with thin but intact bony walls and absence of destructive features, which helps distinguish it from pneumocele and other cystic lesions [8].

Due to its rarity, PSD is often misdiagnosed or incidentally discovered on imaging performed for unrelated complaints. When symptomatic, treatment options range from observation in mild cases to surgical intervention such as sinus obliteration or fenestration for decompression and symptomatic relief.

In our report, the patient was symptomatic during longer flights and did not respond to standard management of barosinusitis that includes avoidance of precipitating pressure changes, topical and systemic decongestants, intranasal corticosteroids, analgesics, and treatment of underlying sinonasal pathology such as septal deviation or ostial obstruction. During ascent and descent in flights, rapid fluctuations in ambient pressure result in transient pressure gradients across the paranasal sinuses. Although this produces barosinusitis in ostial obstruction, altered pressure transmission within an enlarged sinus may stimulate nerve endings in sinus mucosa despite sinus ventilation.

In the present case, carbamazepine resulted in significant pain relief, suggesting the possibility of a neuropathic component to the altitude-related headache. Carbamazepine is used as the first-line drug in treating neuropathic pains like trigeminal neuralgia, atypical facial pain, and post-sinus surgery neuralgia due to its action on voltage-gated sodium channels, reducing ectopic neuronal firing [9,10]. Recognition of neuralgic pain patterns, such as paroxysmal pain, triggerability, minimal radiological disease, or poor response to non-steroidal anti-inflammatory drugs, may help identify patients who could benefit from carbamazepine therapy. Nevertheless, cautious use is warranted due to potential adverse effects, including dizziness, hyponatremia, hepatic dysfunction, and hematological abnormalities, necessitating appropriate patient selection and monitoring [10].

## Conclusions

This case underlines the significance of exploring unusual structural explanations such as PSD in individuals with flight-induced headaches who do not respond to standard treatment for barosinusitis or migraine. PSD of the frontal sinus is a rare and generally undiagnosed anatomical condition that can cause altitude-related headaches, especially during air travel. The considerable clinical improvement seen with carbamazepine suggests a neuropathic pain mechanism caused by barometric pressure variations, most likely due to trigeminal nerve sensitization rather than mucosal inflammation.

While surgical intervention remains the ultimate treatment for symptomatic PSD in some circumstances, pharmacological management with neuropathic pain modulators may provide excellent symptom relief in carefully selected patients. Further research is needed to better understand the pathophysiology of PSD-related pain and to identify the potential of carbamazepine as a treatment option in altitude-induced headache linked with hyper-pneumatized paranasal sinuses. Further research is needed to determine the efficacy, appropriate dose, and safety profile of carbamazepine in this clinical scenario.
